# PDL1 inhibitors may be associated with a lower risk of allograft rejection than PD1 and CTLA4 inhibitors: analysis of the WHO pharmacovigilance database

**DOI:** 10.3389/fimmu.2025.1514033

**Published:** 2025-01-22

**Authors:** Alexandre O. Gérard, Diane Merino, Jonathan Benzaquen, Alexandre Destere, Delphine Borchiellini, Clément Gosset, Fanny Rocher, Marine Andreani, Charles-Hugo Marquette, Henri Montaudié, Milou-Daniel Drici, Antoine Sicard

**Affiliations:** ^1^ Department of Nephrology-Dialysis-Transplantation, Université Côte d’Azur, University Hospital Centre of Nice, Nice, France; ^2^ Department of Clinical Pharmacology, Université Côte d’Azur, University Hospital Centre of Nice, Nice, France; ^3^ Laboratory of Molecular Physio Medicine (LP2M), UMR 7370, CNRS, University Côte d’Azur, Nice, France; ^4^ Molecular and Cellular Pharmacology Institute (IPMC), UMR 7275, CNRS, Université Côte d’Azur, Nice, France; ^5^ Molecular and Cellular Pharmacology Q6 Institute (IPMC), UMR 7275, CNRS, Université Côte d'Azur, Nice, France; ^6^ Department of Clinical Research and Innovation, Department of Medical Oncology, Centre Antoine Lacassagne, Nice, France; ^7^ Department of Dermatology, Université Côte d’Azur, University Hospital Centre of Nice, Nice, France; ^8^ Mediterranean Center for Molecular Medicine (C3M), UMR 1065, INSERM, Université Côte d’Azur, Nice, France

**Keywords:** immune checkpoint inhibitors, allograft rejection, transplantation, pharmacovigilance, oncology

## Abstract

**Background:**

Transplant recipients face increased cancer mortality due to immunosuppressive treatments. Immune checkpoint inhibitors (ICI) have improved survival rates, but data on the use of these agents in transplant recipients is scarce. ICI may trigger allograft rejection, but the absolute risk of AR between the different ICI classes remains to be defined.

**Methods:**

VigiBase^®^ (WHO’s pharmacovigilance database) was queried for reports of AR involving CTLA4, PD1, or PDL1 inhibitors. Disproportionality analysis compares the proportion of reports with a specific adverse drug reaction (ADR) and a given drug to the proportion of reports with the same ADR and other drugs. A lower 95% confidence interval for the Information Component (IC) >0 suggests a signal. The comparative Reporting Odds Ratios (ROR) for AR, between PD1 and PDL1 inhibitors, was calculated.

**Results:**

We gathered 159 AR involving an ICI, especially nivolumab (73, 45.9%), mostly affecting kidneys (87, 54.7%). Median time to onset: 28 days. Fatal outcome: 36 reports (22.6%). ICI were significantly associated with AR: IC=1.7 [1.4;1.9]. Specifically, PD1 inhibitors yielded an IC of 2.0 [1.7;2.2] (152 reports observed compared to 38 expected). By contrast, the IC of PDL1 inhibitors was negative: -2.6 [-6.4;-1.0] (1 observed, 9 expected). The comparative ROR of PD1 compared to PDL1 inhibitors was 33.7 [4.7;240.9] (p=0.0005).

**Conclusions:**

We confirm the association between ICI treatment and AR. Notably, PDL1 inhibitors showed surprisingly low AR reports compared to CTLA4 and PD1 inhibitors. Further prospective studies are warranted to confirm whether PDL1 inhibitors indeed reduce AR risk compared to other ICI.

## 1 Introduction

Solid organ transplant recipients are at heightened risk of death from cancer. Indeed, the immunosuppressive treatment, which is paramount to avoid transplant rejection, heightens the risk of death from cancer among solid organ transplant recipients (1). Yet, evidence-based data regarding their cancer treatment are scarce because transplant recipients are usually excluded from clinical trials, and registries are limited (2, 3).

In recent years, the development of immune checkpoint inhibitors (ICIs), including cytotoxic T-lymphocyte-associated protein 4 (CTLA4) inhibitors and inhibitors of programmed cell death protein 1 or its ligand (PD1, PDL1), has considerably improved the survival of patients with certain advanced cancers (4). These monoclonal antibodies foster immune responses against malignancies, but sometimes lead to off-target immune adverse drug reactions (ADRs) (5). ICI unleash the breaks on the immune system and may trigger allograft rejection (AR) (6, 7).

Based on their similar mechanisms of action, different ICI classes are though to mediate the same effect on AR, however this has never been investigated (8). Besides, although AR associated with ipilimumab and pembrolizumab are mentioned in drug labels from both the Food and Drug Administration (FDA) (9, 10) and the European Medicines Agency (EMA) (11, 12), AR is only addressed in the FDA’s drug label for cemiplimab (13, 14). Similarly, regarding PDL1 inhibitors, AR risk is inconsistently mentioned in the FDA’s drug labels, and does not appear in the EMA’s drug labels. Therefore, we aimed to clarify the association of AR with the different ICI classes, through a disproportionality analysis of the pharmacovigilance database of the World Health Organization (WHO).

## 2 Materials and methods

### 2.1 Database

The Uppsala Monitoring Center (UMC) manages the WHO Safety Database, also known as VigiBase^®^ (15). Since 1967, VigiBase^®^ collects Individual Case Safety Reports from over 172 countries’ national pharmacovigilance networks. These spontaneous reports are submitted after drugs’ marketing, and may be issued by healthcare professionals, patients, and pharmaceutical companies. The anonymity of both patients and reporters is preserved. Each report includes demographic details such as the country of origin, qualifications of the reporter, patient characteristics, information about the drugs involved (for instance indication, start and stop dates, dosage, and any other medications taken simultaneously), and information about ADR (including the effects, seriousness, onset, and outcome of the reaction).

### 2.2 Query

Vigibase^®^ was queried for all reports of the High Level Term (HLT) “Transplant Rejection” involving either the CTLA4 inhibitor (ipilimumab), PD1 inhibitors (cemiplimab, emiplimab, dostarlimab, nivolumab, pembrolizumab, retifanlimab, tislelizumab, toripalimab), or PDL1 inhibitors (atezolizumab, avelumab, durvalumab). In the Medical Dictionary for Regulatory Activities (MedDRA, version 26.1), a HLT is a grouping of related Preferred Terms (PTs) based upon anatomy, pathology, physiology, etiology or function (16). A PT is defined as the distinct descriptor for a single medical concept. For instance, the “Transplant Rejection” HLT includes PTs such as “Kidney transplant rejection”, “Liver transplant rejection” or “Heart transplant rejection” *inter alia*.

Quantitative variables were described in terms of medians with interquartile ranges (IQR) and/or minimum–maximum ranges (min–max). Qualitative variables were described with numbers and proportions.

### 2.3 Disproportionality analysis

Disproportionality analysis is a statistical technique used in pharmacovigilance to identify potential signals regarding ADRs. It consists of comparing the proportion of reports with a specific ADR and a given drug (cases) to the proportion of reports with the same ADR and other drugs (non-cases). If the proportion of cases is higher than that of non-cases, it suggests a possible association between the drug and the ADR.

Disproportionality can be assessed through the Information Component (IC [95% confidence interval]). This measure derives from a Bayesian confidence propagation neural network, and is validated by the Uppsala Monitoring Centre (UMC). The IC compares the observed and expected number of reports for a specific ADR-drug combination. It offers a more accurate detection of potential pharmacovigilance signals, reducing the risk of false positive signals compared to other disproportionality measures. A positive lower end of the 95% confidence interval of the IC is commonly used as the threshold for identifying potential safety signals (see **Supplementary Material**) (17).

We calculated the IC to assess whether transplant rejections (HLT “Transplant Rejection”) are disproportionately reported in association with ICIs. Specifically, we also calculated the IC for the association between each class of ICIs (CTLA4, PD1, or PDL1 inhibitors) and the occurrence of AR. Then, we calculated the comparative reporting odds ratio (ROR [95% confidence interval]) for AR with PD1 inhibitors compared to PDL1 inhibitors. ROR approximates the odds ratio of case-control studies and is specific to case-non-case studies. A ROR > 1 suggests that the ADR is more frequently reported with the drug of interest than with the comparator. The higher the ROR, the more statistically relevant is the potential pharmacovigilance signal. The ROR was expressed as a point estimate with a 95% Confidence Interval, using Woolf’s method (see **Supplementary Material**) (18).

## 3 Results

### 3.1 Characteristics of the reports

**Figure 1:**
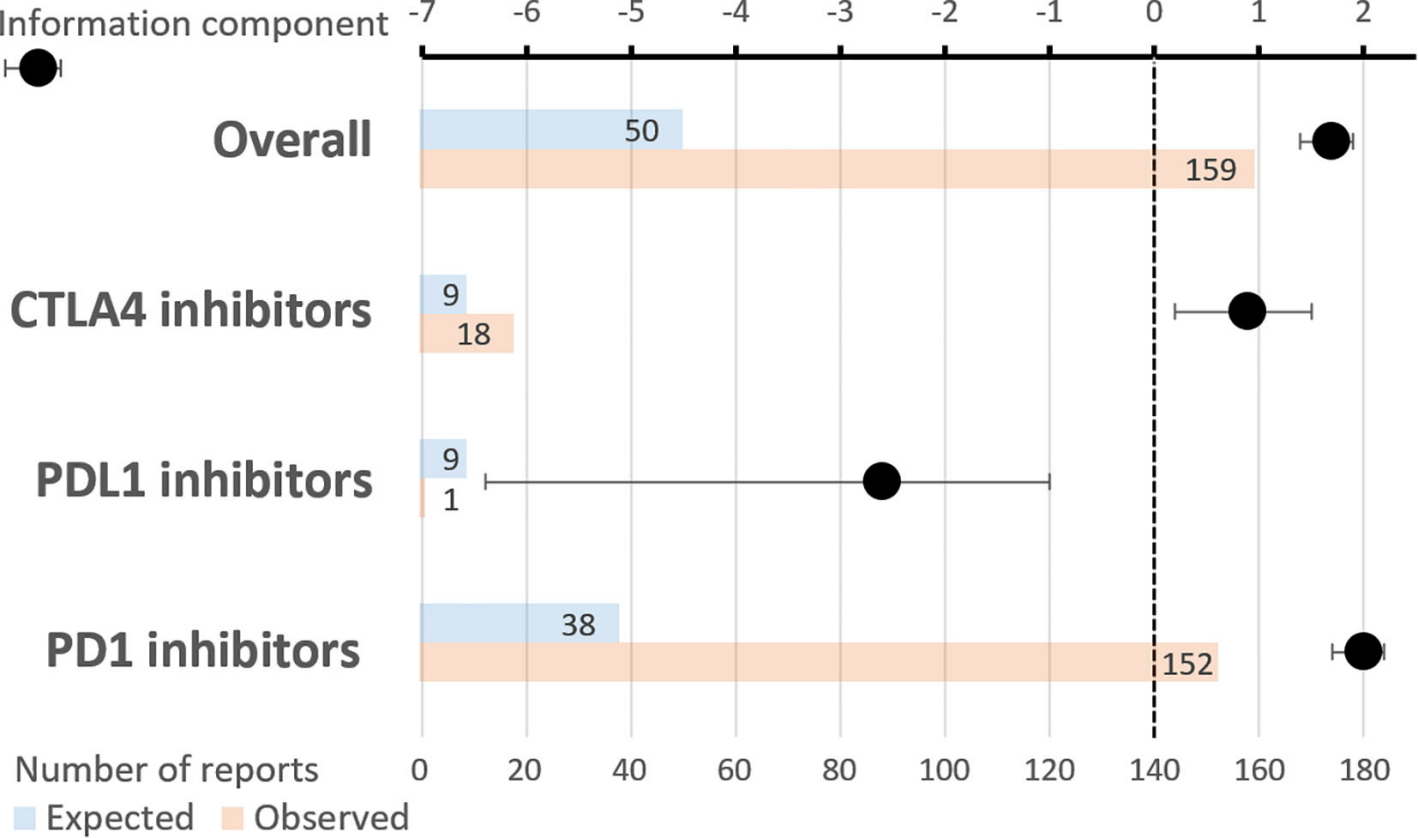
Temporal trends in transplant rejections involving immune checkpoint inhibitors reported to VigiBase. Vertical axis: percentage of the total number of reports. Horizontal axis: year of reporting.

As of 8 February 2024, 159 reports accounting for the HLT “Transplant Rejection” involving an ICI were gathered, accounting for 0.1% of the 171,230 cases reported with ICI in VigiBase^®^. The first case has been reported in 2015 (**Figure 1**). Most patients were male (n=105, 66.0%), with a median age of 63 years (IQR 52-70, min-max 14-85). Most cases were reported in the United States of America (89, 56.0%) and in France (21, 13.2%), mostly by healthcare professionals (152, 95.6%).

The most frequently involved ICIs were PD1 inhibitors, especially nivolumab (n=73, 45.9%) and pembrolizumab (n=62, 39.0%). The CTLA4 inhibitor, ipilimumab, was involved in 18 reports (11.3%). One report (0.6%) involved a PDL1 inhibitor, avelumab. Combined PD1 and CTLA4 inhibitors were used in 12 reports (7.5%). The most co-reported active ingredients were mycophenolic acid (n=31, 10.7%), prednisone (n=28, 10.1%), tacrolimus (n=26, 7.5%), sirolimus (n=17, 5.7%), ciclosporin, and everolimus (n=10 each, 5.0%).

### 3.2 Characteristics of the adverse drug reactions

AR mostly concerned kidney transplants (n=87, 54.7%), then liver transplants (25, 15.7%). The three most frequently co-reported PTs were “malignant neoplasm progression”, “acute kidney injury”, and “intentional product use issue”, with 12 reports each (7.5%).

The median time to onset between ICI introduction and the occurrence of AR was 28 days (IQR 16-60, minimum 11 days, maximum 9 months), corresponding to a median of 1 dose of ICI. A fatal outcome was mentioned in 36 reports (22.6%), mostly in patients with liver transplant rejection (n=15, 41.7% of fatal outcomes). Patients recovered or were recovering from AR in 34 reports (21.4%), and recovered with sequelae in 4 reports (2.5%).

### 3.3 Disproportionality analysis

**Figure 2:**
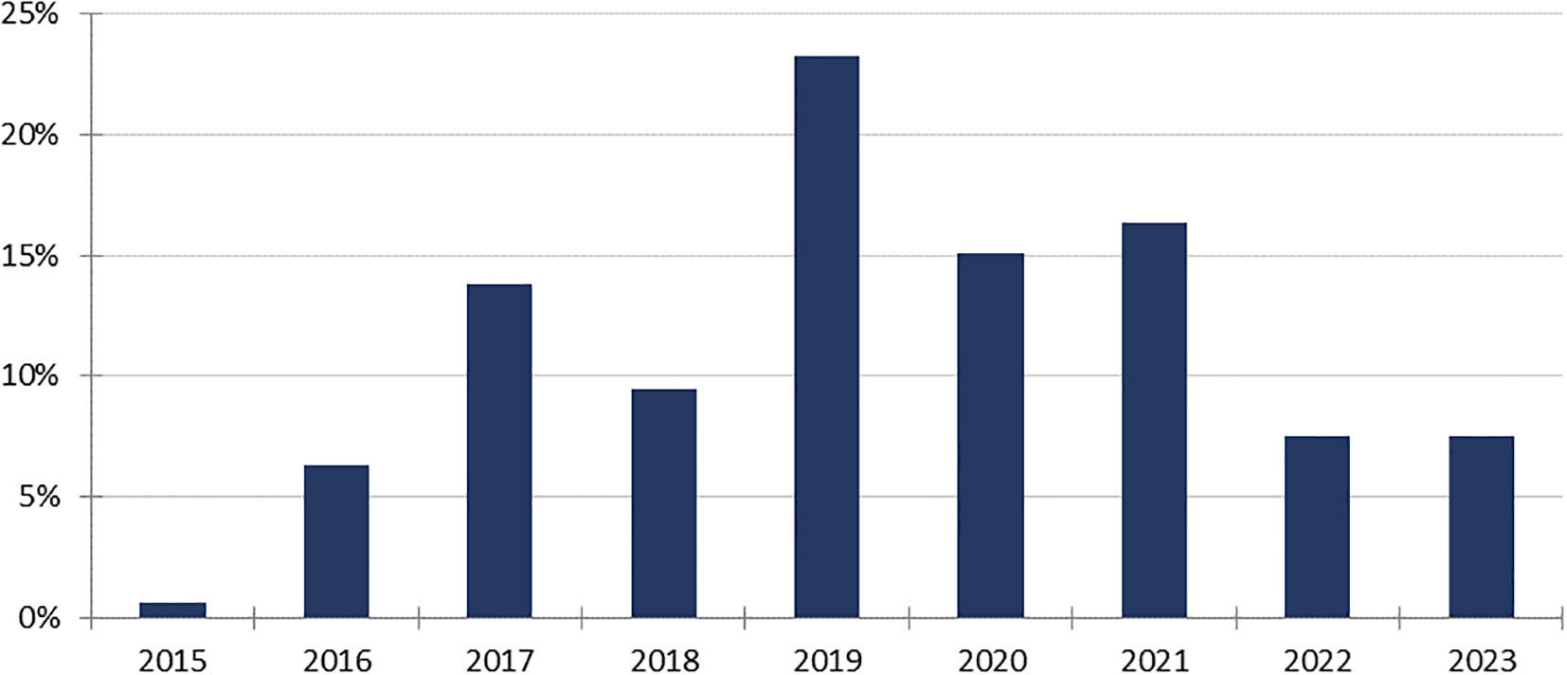
Reports of transplant rejection involving immune checkpoint inhibitors. Upper horizontal axis: information component with its 95% confidence interval. Lower horizontal axis: number of reports (expected and observed). Combined PD1 and CTLA4 inhibitors were used in 12 reports.

As a whole, ICIs were significantly associated with AR, with an IC of 1.7 [1.4;1.9], with 159 reports observed as compared to 50 expected (**Figure 2**). Specifically, PD1 inhibitors exhibited an IC of 2.0 [1.7;2.2], with 152 reports observed as compared to 38 expected. CTLA4 inhibitors showed an IC of 0.9 [0.2;1.5], with 18 reports observed as compared to 9 expected. By contrast, the IC of PDL1 inhibitors did not reach statistical significance: -2.6 [-6.4;-1.0], with 1 report observed as compared to 9 expected. The comparative ROR of PD1 inhibitors, as compared to PDL1 inhibitors, was 33.7 [4.7;240.9] (p=0.0005).

## 4 Discussion

This analysis of real-world data, issued from the world pharmacovigilance database confirms that treatment of solid organ transplant recipients with ICIs is significantly associated with the risk of AR. This result seems plausible, due to the mechanisms of action of ICIs (19). It is also consistent with the results of previous studies (6, 7, 20). Our results suggest that AR occurs at an early stage of treatment and is associated with poor outcomes. Most reports or AR occurred in kidney transplant recipients, mostly because kidney transplantation is more prevalent than other solid organ transplantations. Moreover, clinicians might be less reluctant to use ICIs on kidney transplant recipients, because renal replacement therapy by dialysis is available. By contrast, liver (or heart) transplant rejection can be life-threatening on a short term.

In the literature, almost all reported cases occur in patients who have been transplanted for more than one year, and sometimes for more than 10 years, before the initiation of an ICI (20). This may be because clinicians are aware of the increased risk of rejection in the early post-transplantation period. Moreover, most patients probably undergo thorough screening for potential malignancies before being transplanted, so that it is rarely required to treat advanced cancer shortly after transplantation. According to the literature, when AR occurs, most patients receive high-dose corticosteroids, sometimes supplemented with intravenous immunoglobulins (in the case of antibody-mediated rejection) (21). For some patients, tacrolimus and/or mycophenolate are initiated or their dose are increased (20). Data from a systematic review report a mortality rate of 57.8% with a longer follow-up duration (median overall survival: 36 weeks) (7). Most deaths were due to cancer progression, although AR significantly impacted survival, particularly in liver recipients.

Interestingly, only one case of AR involving PDL1 inhibitors was reported in the WHO pharmacovigilance database, which is surprisingly low. Moreover, whereas CTLA4 and PD1 inhibitors were associated with statistically significant safety signals for AR in VigiBase^®^, no signal was found with PDL1 inhibitors. The IC for PDL1 inhibitors was even negative, as 9 reports of ARs were expected considering the total number of reports with this class and with this ADR. Our findings are supported by a multicenter retrospective study, which focused on the safety and efficacy of ICIs in kidney transplant recipients. Among the 6 patients treated with PDL1 inhibitors, none developed AR, while almost half of the patients treated with PD1 inhibitors did (21). This class seemed associated with a lower risk of rejection, though the number of patients in this non-randomized cohort was too limited to draw definite conclusions.

Debate is still ongoing regarding the different safety profiles of PD1 and PDL1 inhibitors. Both molecules are involved in immune tolerance in transplant recipients (22). Therefore, the potentially lowered risk of AR in patients treated with PDL1 inhibitors might be underpinned by the fact that few lymphocytes are trafficking in the transplanted organ at a baseline state. Hence, PD1 inhibitors could potentially activate a larger proportion of lymphocytes overall, while PDL1 inhibitors may primarily activate lymphocytes at the tumor site, where PDL1 expression is significantly higher than in the graft (23). Another hypothesis is that the intensity of checkpoint inhibition may vary depending on the chosen class. This suggests a potential interest of therapeutic drug monitoring for ICIs, to reduce the risk of overexposure in at-risk patients (24).

Besides, PDL1 inhibitors do not disrupt the interaction between PD1 and PDL2, unlike PD1 inhibitors. As a result, the PDL2 pathway remains efficient in patients receiving PDL1 inhibitors, as a potential protective mechanism for the transplant (25). In fact, PDL2 is expressed on the human kidney and helps to regulate CD8 proliferation, while promoting Treg functions (26–30). From a different perspective, belatacept, a costimulation blocker targeting the CTLA4 pathway, is increasingly used in transplant recipients. A deeper understanding of the physiology of immune checkpoint blockade could, therefore, clear the path for harnessing these pathways to enhance allograft tolerance.

However, it is necessary to guard against overly speculative assertions. In fact, this study cannot definitely conclude that PDL1 inhibitors decrease the risk of AR, as compared to other ICIs. Indeed, limitations inherent to spontaneous reporting systems and pharmacovigilance approaches are hindering factors. Lacking data are frequent, preventing thorough causality assessment for each reported case of AR. Furthermore, we do not have access to the transplant biopsies and the details on the management of AR. The WHO safety database does not provide data regarding the interval between transplantation and the initiation of an ICI. The lack of case validation can induce false positives (31). Several confounding factors could not be addressed either, such as the indications of the treatment with ICIs, or the overall immunological risk status of the patients. Then, the available data do not allow for a distinction between cancer-related mortality and the direct consequences of rejection. The number of reports is low for some classes, with, for instance, only one report of AR involving PDL1 inhibitors in the WHO safety database. This may be due to PD-L1 inhibitors being less frequently used than other classes of ICIs. However, the disproportionality approach aims at mitigating the possible impact of varying prescribing rates (32, 33). In fact, when a drug is prescribed in a larger number of patients, the occurrence of all ADRs is expected to increase. Thus, the disproportionate reporting of a given ADR does not directly depend on the number of patients exposed to the drug. Anyway, reporting rates cannot be interpreted as incidence rates, hence the need for further confirmatory studies.

All in all, our exploratory study paves the way to further prospective studies investigating whether PDL1 inhibitors decrease the risk of AR compared to other classes of ICIs. Besides, the underlying mechanisms involved in this discrepancy warrant further investigation. If these data are confirmed, PDL1 inhibitors could become the preferred class of immune checkpoint inhibitors in solid organ transplant recipients.

## Data Availability

The datasets generated during and/or analyzed during the current study are not publicly available because data are owned by UMC, who manages VigiBase^®^, but are available from the corresponding author on reasonable request. Requests to access these datasets should be directed to pharmacovigilance@chu-nice.fr.
