# Supplementary material for: PDL1 inhibitors may be associated with a lower risk of allograft rejection than PD1 and CTLA4 inhibitors: analysis of the WHO pharmacovigilance database
Source: Front Immunol. 2025 Jan 22;16:1514033. doi: 10.3389/fimmu.2025.1514033 (PMC11794220; doi:10.3389/fimmu.2025.1514033)
Supplement: Supplementary file 1 [file DataSheet1.docx]

Supplementary Material

The formulas for Information Component (IC) and Reporting Odds Ratio (ROR) are described below.

|  | Transplant rejection | Other adverse drug reactions |
| --- | --- | --- |
| Selected active ingredient | A | B |
| All other active ingredients | C | D |

IC = log2 [(A + 0.5)/(*N*_expected_ + 0.5)] where *N*_expected_ = [(A + B) × (A + C)]/(A + B + C + D)

ROR = (A/C)/(B/D)
